# Acute and subacute oral toxicity assessment of Gancao Xiexin decoction in Sprague-Dawley rats

**DOI:** 10.3389/fphar.2022.1078665

**Published:** 2023-01-10

**Authors:** Li Ru, Ruotong Liu, Haoyu Xing, Yueming Yuan, Zheng Yuan, Zhiyong Xu, Qin Xu, Jianping Song, Xiaobo Li

**Affiliations:** ^1^ Artemisinin Research Center, Guangzhou University of Chinese Medicine, Guangzhou, China; ^2^ Sci-Tech Industrial Park, Guangzhou University of Chinese Medicine, Guangzhou, China; ^3^ The First Affiliated Hospital of Guangzhou University of Chinese Medicine, Guangzhou, China

**Keywords:** Gancao Xiexin decoction, acute toxicity, subacute toxicity, rats, safety

## Abstract

Gancao Xiexin decoction (GCXXD), a well-known classic traditional Chinese medicine prescription, is used to treat various oral ulcers, Behcet disease, gastrointestinal ulcers, etc. However, there is very little information on its safety. This study aimed to investigate the acute and subacute oral toxicity of GCXXD in Sprague-Dawley rats. In the acute toxicity study, rats were orally administered 10 g/kg GCXXD three times a day. Clinical signs of abnormality and mortality were observed daily for 14 days. In the subacute toxicity study, rats were orally administered 0, 1.47, 3.83, or 10 g/kg GCXXD for 28 days. The rats’ clinical signs, body weight, food consumption, hematological and biochemical parameters, bone marrow smear, organ index, and pathological morphology were analyzed. The acute toxicity study showed that GCXXD is safe in rats without any obvious toxicity *via* an oral dose of 30 g/kg/day (3 × 10 g/kg). After 28 days of administration, slightly decreased RBC, HGB, and HCT were observed in female rats at 10 g/kg, suggesting that repeated doses of high-dose GCXXD may cause mild anemia in female rats. The no-observed-adverse-effect level (NOAEL) and lowest-observed-adverse-effect level (LOAEL) of oral administration of GCXXD for 28 days in rats are considered to be 3.83 g/kg and 10 g/kg, respectively. Long-term toxicity studies are recommended to strengthen the findings.

## 1 Introduction

Traditional Chinese medicine (TCM) is the quintessence of Chinese culture, with thousands of years of clinical application history in China. The field of TCM is also a vast and untapped resource for modern medicine (Wang et al., 2018). Recently, the World Health Organization (WHO) first included traditional medicine disorders and patterns derived from ancient Chinese medicine that is commonly used in China, Japan, Korea, and elsewhere around the world in the 11th revision of the International Classification of Diseases (ICD) (Xu et al., 2020). As one of the major aspects of TCM, herbal medication has played significant roles in treating traditional diseases and developing modern drugs (Yuan et al., 2016). The use of herbal medicine and its supplements has increased dramatically worldwide, and about 80% of the world’s population relies on herbal medicinal products to improve their health (WHO, 2008). However, many commonly used herbal formulas have not described their ingredients, efficacy, and safety in detail, which may lead to adverse reactions and drug interactions in clinical practice (Efferth and Kaina, 2011; Calapai, 2012; Zeng et al., 2017). The adverse effects of herbal medicine have been reported in recent years, such as hepatotoxicity, nephrotoxicity, and cardiotoxicity, and their toxicity has roused widespread concern (Ekor, 2014; Hudson et al., 2018; Farzaei et al., 2020). Therefore, in consideration of the extensive use of herbal formulas, toxicological evaluations are much needed to ensure their quality and safety, so as to protect public health.

Gancao Xiexin decoction (GCXXD) is one of the classic prescriptions in ancient China, which originated from the “Treatise on Febrile Diseases” by Zhongjing Zhang, a medical sage in the Han Dynasty (Cai ang Gan, 2022). GCXXD contains six herbal materials (see Table 1 for details), including *Glycyrrhiza uralensis* Fisch. (Chinese name: Gancao), *Scutellaria baicalensis* Georgi (Chinese name: Huangqin), *Pinellia ternata* (Thunb.) Breit. (Chinese name: Banxia), *Zingiber officinale* Rosc. (Chinese name: Ganjiang), *Coptis chinensis* Franch. (Chinese name: Huanglian), and *Ziziphus jujuba* Mill. (Chinese name: Dazao). GCXXD has been recorded in ancient books to treat spleen and stomach weakness and abdominal distention syndrome with mixed cold and heat. Currently, it is widely used in modern clinical practice for various oral ulcers, Behcet disease, gastrointestinal ulcers, and also has a certain effect on gynecological diseases and eczema (Shi and Zhang, 2022). It has been reported that GCXXD has pharmacological effects, such as regulating gastric acid secretion, promoting wound healing, anti-inflammation, and regulating immunity (Hu et al., 2022).

**TABLE 1:** Information of components in GCXXD.

Latin name	English name	Chinese name	Plant part	Amount (g)
*Glycyrrhiza uralensis* Fisch.	Liquorice Root	Gancao	Root and rhizome	12 (25.00%)
*Scutellaria baicalensis* Georgi	Baical Skullcap Root	Huangqin	Root	9 (18.75%)
*Pinellia ternata* (Thunb.) Breit.	Pinellia Tuber	Banxia	Tuber	9 (18.75%)
*Zingiber officinale* Rosc.	Dried Ginger	Ganjiang	Rhizome	9 (18.75%)
*Coptis chinensis* Franch.	Coptis Root	Huanglian	Rhizome	3 (6.25%)
*Ziziphus jujuba* Mill.	Jujube	Dazao	Fruit	6 (12.50%)
Total amount				48 (100.00%)

Recently, to encourage the development and research of classic prescriptions and further explore the scientific value of TCM, the China Administration of Traditional Chinese Medicine screened out 100 ancient classic prescriptions as critical development objects from more than 100,000 prescriptions recorded in 103 medical books. GCXXD is the 11th prescription in the catalogue of these 100 classic prescriptions, so it has broad development and application prospects. Although there are many researches on GCXXD, they mainly focus on the clinical therapeutic effect (Wu et al., 2021; Yan et al., 2021; Zhang et al., 2021), animal efficacy (Zhang et al., 2021; Li et al., 2022; Luo et al., 2022), or chemical composition analysis (Shen et al., 2021; He et al., 2022; Tang et al., 2022). Little published information is available about its safety. Therefore, we assessed the acute and subacute toxicity of GCXXD in rats in this study. The results could provide a scientific basis for its safe clinical application.

## 2 Materials and methods

### 2.1 Test product and preparation

GCXXD (batch number: 190516) extract was produced and provided by the Modern Chinese Patent Medicine Engineering Research and Development Center of Guangzhou University of Traditional Chinese Medicine Sci-tech Industrial Park Co., Ltd. (Guangzhou, China). All the raw herbs in GCXXD were purchased from Zisun Chinese Pharmaceutical Co., Ltd. (Guangzhou, China) and complied with the quality standards of Chinese Pharmacopoeia (version 2015).

The preparation process of GCXXD extract complied with the following standard operating procedures. 12 g *Glycyrrhiza uralensis* Fisch., 9 g *Scutellaria baicalensis* Georgi, 9 g *Pinellia ternata* (Thunb.) Breit., 9 g *Zingiber officinale* Rosc., 3 g *Coptis chinensis* Franch., and 6 g *Ziziphus jujuba* Mill. were put into a round-bottom flask, and then added 2000 mL to soak for 30 min, then heated and refluxed for 2 h. The extracted solution filtered by gauze was concentrated through rotary evaporation under vacuum at 80°C. Finally, the concentrated solution was dried at 70°C under reduced pressure to obtain GCXXD powder (yield: 35.3%). Referring to the quantification methods in the Chinese Pharmacopoeia (version 2015), the quality control of GCXXD extract was performed using high-performance liquid chromatography (HPLC) by detecting glycyrrhizin, baicalin, berberine hydrochloride, baicalein, ammonium glycyrrhizinate, 6-gingerol. GCXXD suspension was freshly prepared with purified water daily prior to animal dosing.

### 2.2 Animals

Male and female specific pathogen-free Sprague-Dawley (SD) rats (6–8 weeks old, weighing 200–250 g) were purchased from Beijing Vital River Laboratory Animal Technology Co., Ltd. (Beijing, China). The rats were housed in metal cages (controlled room temperature 20°C–26°C, relative humidity 40%–70%, and 12 h light/dark reverse cycle) with free access to purified water and standard rodent chow diet. In accordance with the Principles of Good Laboratory Practice (GLP) from the National Medical Products Administration (NMPA), China, the animal experiments were conducted in the laboratory animal room of the New South Center of Safety Evaluation for Drugs of Guangzhou University of Chinese Medicine (China Animal Use License No.: SYXK (Guangdong) 2018–0014), and were approved by its ethics committee for laboratory animal care and use.

### 2.3 Acute toxicity study

20 rats were randomly divided into 2 groups of 10 animals (5 females and 5 males): a control group (water) and a dose group (30 g/kg). The concentration of GCXXD was 0.5 g/mL, the maximum concentration that could be administered to the rats *via* gavage. For 16 h before the experiment, the rats had access to water but no food. GCXXD or water was orally administered to rats 3 times, with an interval of 6 h, and the dose volume was 20 mL/kg body weight. The rats were carefully observed for mortality and any clinical signs of abnormality for 6 h after each administration on Day 0, and then twice daily for 14 days. The body weight was also noted on Day 0, 1, 7, and 14. At the end of the experiment, the animals were sacrificed to examine gross anatomy.

### 2.4 Subacute toxicity study

120 rats were randomly assigned to 4 groups of 30 animals (15 females and 15 males): a control group (water) and GCXXD 1.47, 3.83, 10 g/kg groups (defined as low-dose, medium-dose, high-dose groups, respectively). The dose design of GCXXD was based on the finding of the acute toxicity study and its proposed adult daily dose (11 g/day). According to the equivalent body surface area conversion method, if the adult weight is 60 kg, the above clinical dose in rats was approximately equivalent to 1.1 g/kg. The high dose was set at 10 g/kg, obtained by multiplying the maximum concentration of GCXXD (0.5 g/mL) that can be prepared and the maximum administration volume (20 ml/kg) suitable for long-term gastric perfusion. The medium and low doses were 3.83 and 1.47 g/kg according to the principle of 2.61 times equal ratio, of which the low dose was slightly higher than the clinical equivalent dose. All animals underwent oral gavage (20 mL/kg body weight) once daily for 28 days, followed by a recovery period of 2 weeks. The rats were observed twice daily for clinical signs, and their body weights and food consumption were recorded once weekly.

After treatment for 28 days, twenty rats (10 females and 10 males) of each group fasted for about 16 h, blood samples were collected *via* the abdominal aorta after intravenous injection of pentobarbital sodium (30 mg/kg), and then suffered from acute hemorrhage and euthanasia. Of the remaining rats, ten animals (5 females and 5 males) of each group were euthanized as above at the end of recovery period. About 1.9 mL of blood was collected in anticoagulant tube containing ethylenediamine tetraacetic acid dipotassium and measured using an ADVIA 2120i automatic blood analyzer (Siemens, Germany) for the following hematological parameters: White blood cell count (WBC), red blood cell count (RBC), hemoglobin (HGB), hematocrit (HCT), mean corpuscular volume (MCV), mean corpuscular hemoglobin (MCH), mean corpuscular hemoglobin concentration (MCHC), reticulocyte count (Ret), platelet count (PLT), lymphocyte (LYM), monocyte (MON), neutrophil (NEU), eosinophils (EOS), and basophils (BAS). About 1.8 mL of blood was collected in anticoagulant tube containing sodium citrate for coagulation testing. After separating plasma from blood at 3,000 rpm for 10 min, prothrombin time (PT), activated partial thromboplastin time (APTT), thrombin time (TT), and fibrinogen concentration (Fbg) for blood coagulation were measured using a CA1500 automatic coagulometer (Sysmex, Japan). About 3 mL of blood in tube without anticoagulant was centrifuged (3,000 rpm, 10 min) to afford serum for the biochemical examinations. The serum concentrations of alanine aminotransferase (ALT), aspartate aminotransferase (AST), creatinine (CRE), gamma-glutamyltransferase (GGT), alkaline phosphatase (ALP), urea nitrogen (UREA), total bilirubin (TBIL), creatine kinase (CK), total protein (TP), albumin (ALB), glucose (GLU), triglyceride (TC), and total cholesterol (CHOL) were assessed using a 7080 automatic biochemical analyzer (Hitachi, Japan). The sodium (Na^+^), chloride (Cl^−^), and potassium (K^+^) concentrations were tested by an EasyLyte PLUS Na^+^\K^+^\Cl^−^ electrolyte analyzer (Medica, USA).

The brain, liver, spleen, adrenal gland, epididymis, uterus, heart, kidney, testis, ovary, lung, and thymus were weighed to calculate organ index (absolute organ weight/body weight). Bone marrow of the femur was taken for bone marrow smear and processed with Wright’s staining. The tissues and organs (brain, liver, spleen, adrenal gland, epididymis, uterus, heart, kidney, testis, ovary, lung, thymus, hypophysis, spinal cord, thyroid, parathyroid, salivary gland, harderian gland, eyeball, trachea, esophagus, etc.) were preserved in 10% neutral buffered formalin and evaluated histopathologically.

### 2.5 Statistical analysis

Statistical Product and Service Solutions (SPSS) version 19.0 statistical software was used for the analysis. Data were expressed as mean and standard deviation (means ± SD). If the variance was homogeneous, the difference between groups was evaluated by the one-way analysis of variance (ANOVA) test. If not, the Kruskal-Wallis non-parametric test was used. When significance was noted in the ANOVA test, the data were analyzed by the multiple comparison procedure of the Dunnett’s test. *p* ≤ 0.05 was considered statistically significant.

## 3 Results

### 3.1 Acute oral toxicity in rats

**FIGURE 1:**
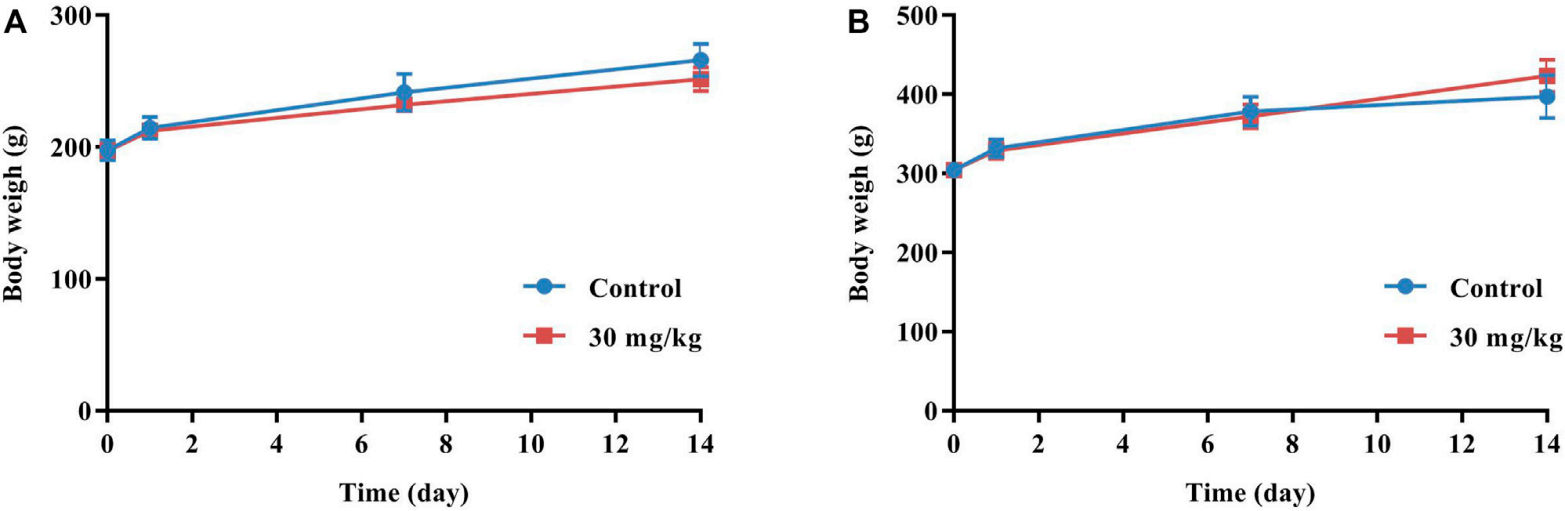
Mean body weights of female **(A)** and male **(B)** rats in acute toxicity study.

No animal mortality occurred, and no abnormal signs were observed throughout the experiment. As shown in Figure 1, the body weights of the control group and the dose group were both increased, and there was no significant difference between the average body weight of the two groups. In addition, no gross pathological signs were found in any organs of all rats during necropsy.

### 3.2 Subacute oral toxicity in rats

#### 3.2.1 Clinical signs, body weight, and food consumption

There were no animal deaths or abnormal clinical signs related to the administration of GCXXD throughout the study. All the rats in low-, medium- and high-dose groups and control group were in good condition, and no obvious abnormalities were observed in fur color, behavior, feeding, drinking, breathing, urine, feces, etc.

**FIGURE 2:**
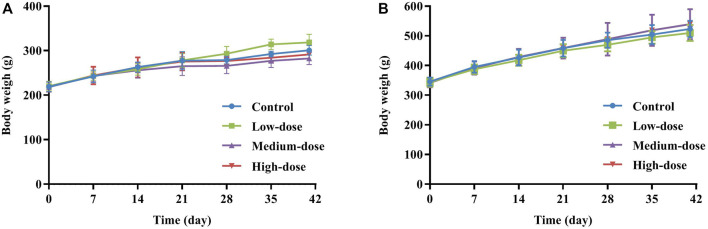
Mean body weights of female **(A)** and male **(B)** rats in subacute toxicity study.

As presented in Figure 2, all animals in each group showed steady body weight gain throughout the study period, with no significant difference in body weight between control and treated rats at each measurement time point. Similarly, since there were no differences in food consumption in any group during the administration and recovery periods, GCXXD had no adverse effect on animal food consumption.

#### 3.2.2 Hematology

The results of hematological parameters measured at the end of administration are summarized in Table 2. The MCH of female rats in the low-dose group decreased significantly, which was not considered toxicologically meaningful due to the absence of a dose-response relationship. Significant decreases in HGB and HCT were found in females at high dose. The corresponding RBC also decreased, even though there was no statistical difference. Combined with the internal hematological data of normal rats from the New South Center of Safety Evaluation for Drugs of Guangzhou University of Chinese Medicine, the HGB, HCT, and RBC ranges of normal female rats were 13.2–15.7 g/dL, 39.0%–47.8%, and 7.08–8.75×10^12^/L, respectively. While the average HGB, HCT, and RBC of female rats in the high-dose group were 12.7 g/dL, 39.0%, and 6.89×10^12^/L, respectively, which were slightly decreased. Therefore, we presumed that high doses of GCXXD might cause mild anemia in female rats. The above anomalous hematological parameters returned to normal values after 14 days of recovery.

**TABLE 2:** Hematological parameters of rats after 28 days of administration with GCXXD.

Parameters	Female (*n* = 10)				Male (*n* = 10)			
	Control	Low-dose	Medium-dose	High-dose	Control	Low-dose	Medium-dose	High-dose
RBC (10^12^/L)	7.26 ± 0.36	7.34 ± 0.35	7.40 ± 0.32	6.89 ± 0.22	7.98 ± 0.28	8.02 ± 0.43	7.82 ± 0.46	7.82 ± 0.20
HGB (g/dL)	13.8 ± 0.5	13.3 ± 0.6	13.7 ± 0.6	12.7 ± 0.4*	14.9 ± 0.4	14.6 ± 0.4	14.3 ± 0.5	14.2 ± 0.5
HCT (%)	41.7 ± 1.2	40.9 ± 2.1	41.6 ± 1.5	39.0 ± 1.3*	44.4 ± 1.5	44.9 ± 1.3	43.8 ± 1.7	43.7 ± 2.2
MCV (fL)	57.6 ± 1.5	55.8 ± 1.4	56.3 ± 1.5	56.6 ± 1.1	55.6 ± 0.9	56.0 ± 2.0	56.0 ± 2.8	55.9 ± 3.1
MCH (pg)	19.0 ± 0.3	18.2 ± 0.4*	18.5 ± 0.5	18.5 ± 0.4	18.6 ± 0.5	18.2 ± 0.7	18.3 ± 0.8	18.1 ± 0.6
MCHC (g/dL)	32.9 ± 0.8	32.6 ± 0.6	33.0 ± 0.3	32.7 ± 0.5	33.5 ± 0.6	32.6 ± 0.3	32.7 ± 0.4	32.4 ± 0.7
PLT (10^9^/L)	1103.4 ± 104.6	1059.0 ± 62.2	1073.4 ± 90.5	1119.6 ± 67.1	1086.0 ± 93.1	1146.6 ± 149.7	1114.0 ± 122.4	1174.0 ± 184.0
Ret (%)	3.4 ± 0.5	3.1 ± 0.5	3.2 ± 0.4	3.7 ± 0.9	3.0 ± 0.3	3.4 ± 0.4	2.8 ± 0.3	3.5 ± 0.3
WBC (10^9^/L)	4.31 ± 0.86	3.28 ± 0.60	5.64 ± 4.11	5.10 ± 2.36	6.18 ± 2.19	7.15 ± 1.63	7.48 ± 3.41	5.62 ± 2.64
NEU (%)	12.1 ± 2.6	15.2 ± 2.6	21.0 ± 15.2	22.1 ± 6.8	18.6 ± 4.2	14.7 ± 4.4	15.7 ± 3.9	17.4 ± 1.7
LYM (%)	84.5 ± 2.6	79.4 ± 2.9	75.7 ± 15.3	74.7 ± 7.2	77.6 ± 4.9	82.1 ± 5.2	81.4 ± 4.2	79.3 ± 1.6
MON (%)	2.0 ± 0.3	3.1 ± 1.3	2.0 ± 0.6	1.6 ± 0.4	2.0 ± 0.4	2.2 ± 1.4	1.9 ± 0.7	1.8 ± 0.3
BAS (%)	1.4 ± 0.1	2.0 ± 0.3	1.2 ± 0.4	1.4 ± 0.3	1.6 ± 0.8	0.9 ± 0.2	0.9 ± 0.4	1.3 ± 0.5
EOS (%)	0.1 ± 0.1	0.3 ± 0.1	0.2 ± 0.1	0.2 ± 0.1	0.1 ± 0.0	0.1 ± 0.1	0.1 ± 0.2	0.2 ± 0.2

*Indicates *p ≤* 0.05 as compared to control group.

In female rats, the APTT decreased significantly at medium dose (Table 3), which was likely toxicologically irrelevant since it was not dose-dependent. The other three coagulation parameters (PT, TT, and Fbg) did not show significant alters.

**TABLE 3:** Coagulation parameters of rats after 28 days of administration with GCXXD.

Parameters	Female (*n* = 10)				Male (*n* = 10)			
	Control	Low-dose	Medium-dose	High-dose	Control	Low-dose	Medium-dose	High-dose
PT (sec)	8.1 ± 0.3	7.9 ± 0.3	8.3 ± 0.5	8.3 ± 0.2	11.0 ± 0.7	10.8 ± 1.7	10.6 ± 0.9	10.1 ± 0.3
APPT (sec)	11.1 ± 1.0	11.6 ± 2.6	14.1 ± 1.2*	14.0 ± 2.0	21.4 ± 1.5	18.9 ± 4.9	21.2 ± 2.3	19.3 ± 1.2
TT (sec)	52.1 ± 1.9	48.2 ± 4.2	51.4 ± 2.0	48.8 ± 1.0	57.6 ± 3.8	57.8 ± 4.8	57.3 ± 3.0	55.8 ± 4.1
Fbg (g/L)	1.8 ± 0.1	1.8 ± 0.2	1.9 ± 0.4	1.8 ± 0.2	2.2 ± 0.1	2.2 ± 0.4	2.2 ± 0.2	1.9 ± 0.1

*Indicates *p ≤* 0.05 as compared to control group.

#### 3.2.3 Serum biochemistry

Table 4 presents the result of serum biochemical parameters determined at the end of administration. The K^+^ of female rats in the medium-dose group was significantly reduced, which was not considered to be related to toxicity due to the lack of dose correlation. The male rats at high dose had a lower Cl^−^, which was supposed to be toxicologically irrelevant because the individual Cl^−^ values within the historical value range of normal rats (99.3–112.3 mmol/L). A significant increase was noted in GGT of female rats at high dose. Considering that no abnormality was observed in the corresponding liver function indicators (ALT, AST, etc.) and liver pathology, and the individual GGT values were also within the historical value range of normal rats (0–1.64 U/L), so it may have no toxicological meaning.

**TABLE 4:** Biochemical parameters of rats after 28 days of administration with GCXXD.

Parameters	Female (*n* = 10)				Male (*n* = 10)			
	Control	Low-dose	Medium-dose	High-dose	Control	Low-dose	Medium-dose	High-dose
ALT (U/L)	22.48 ± 9.35	15.69 ± 1.89	15.08 ± 3.91	19.23 ± 4.69	24.57 ± 5.06	21.29 ± 5.77	20.91 ± 1.62	25.05 ± 3.79
AST (U/L)	101.3 ± 10.1	84.7 ± 14.0	100.7 ± 12.2	108.9 ± 7.2	115.1 ± 17.9	103.4 ± 20.0	100.7 ± 23.9	113.9 ± 18.3
ALP (U/L)	81.61 ± 18.54	63.93 ± 11.49	90.72 ± 27.35	83.62 ± 22.55	153.00 ± 13.87	138.39 ± 15.94	133.88 ± 20.97	134.91 ± 29.59
CRE (μmol/L)	27.6 ± 2.1	26.3 ± 3.5	30.4 ± 4.7	29.5 ± 5.7	26.2 ± 2.8	23.5 ± 3.1	25.0 ± 4.1	24.5 ± 3.3
TBIL (μmol/L)	1.1 ± 0.5	1.1 ± 0.3	1.7 ± 1.5	1.0 ± 0.2	1.7 ± 1.0	1.4 ± 1.0	1.1 ± 0.5	2.0 ± 1.2
GGT (U/L)	0.13 ± 0.18	0.07 ± 0.10	0.48 ± 0.25	0.79 ± 0.34*	0.05 ± 0.11	0.10 ± 0.16	0.16 ± 0.15	0.10 ± 0.12
UREA (mmol/L)	6.42 ± 0.82	6.80 ± 0.75	6.70 ± 0.59	6.31 ± 1.19	6.26 ± 0.22	6.70 ± 1.01	5.91 ± 1.25	6.35 ± 0.35
TP (g/L)	56.87 ± 3.99	56.65 ± 1.93	54.97 ± 4.23	55.98 ± 1.76	50.65 ± 2.26	50.35 ± 2.60	51.11 ± 0.36	49.41 ± 1.60
ALB (g/L)	40.78 ± 3.04	41.07 ± 1.49	39.59 ± 3.37	40.37 ± 1.84	35.93 ± 0.75	35.42 ± 1.30	36.08 ± 0.61	35.51 ± 0.74
CK (U/L)	435.1 ± 111.0	305.0 ± 102.8	376.6 ± 96.0	366.2 ± 46.1	371.3 ± 74.3	325.9 ± 92.4	368.5 ± 94.6	383.0 ± 134.6
GLU (mmol/L)	7.03 ± 0.44	6.33 ± 0.54	6.18 ± 0.72	6.29 ± 0.27	6.67 ± 1.14	6.83 ± 0.87	6.38 ± 0.71	7.78 ± 2.05
CHOL (mmol/L)	1.60 ± 0.23	1.57 ± 0.22	1.59 ± 0.45	1.69 ± 0.18	1.35 ± 0.16	1.39 ± 0.31	1.51 ± 0.16	1.35 ± 0.23
TG (mmol/L)	0.37 ± 0.09	0.37 ± 0.08	0.32 ± 0.07	0.31 ± 0.05	0.30 ± 0.04	0.35 ± 0.07	0.38 ± 0.11	0.45 ± 0.13
Na^+^(mmol/L)	140.5 ± 0.8	140.3 ± 0.7	140.5 ± 1.5	139.5 ± 1.6	141.1 ± 1.5	140.7 ± 1.0	141.2 ± 0.5	139.4 ± 1.3
K^+^ (mmol/L)	4.2 ± 0.2	4.2 ± 0.0	3.8 ± 0.2*	4.0 ± 0.3	4.5 ± 0.2	4.6 ± 0.4	4.5 ± 0.2	4.7 ± 0.5
Cl^−^ (mmol/L)	107.9 ± 1.8	107.2 ± 1.2	107.5 ± 0.4	106.6 ± 1.7	107.8 ± 1.2	107.9 ± 1.2	107.8 ± 0.5	104.7 ± 1.6*

*Indicates *p ≤* 0.05 as compared to control group.

#### 3.2.4 Bone marrow smear, organ index, and histopathology

Through microscopic examination of bone marrow staining smears of rats in the control and high-dose groups at the end of administration, it was found that the proportion of various cells in bone marrow of all rats was reasonable, the morphology of various cells was normal, and the hematopoietic function of bone marrow was not changed.

No remarkable gross pathological changes were detected in rats of each group at the end of administration and recovery period. Similarly, there was no significant difference in organ index between control and treated rats.

**FIGURE 3:**
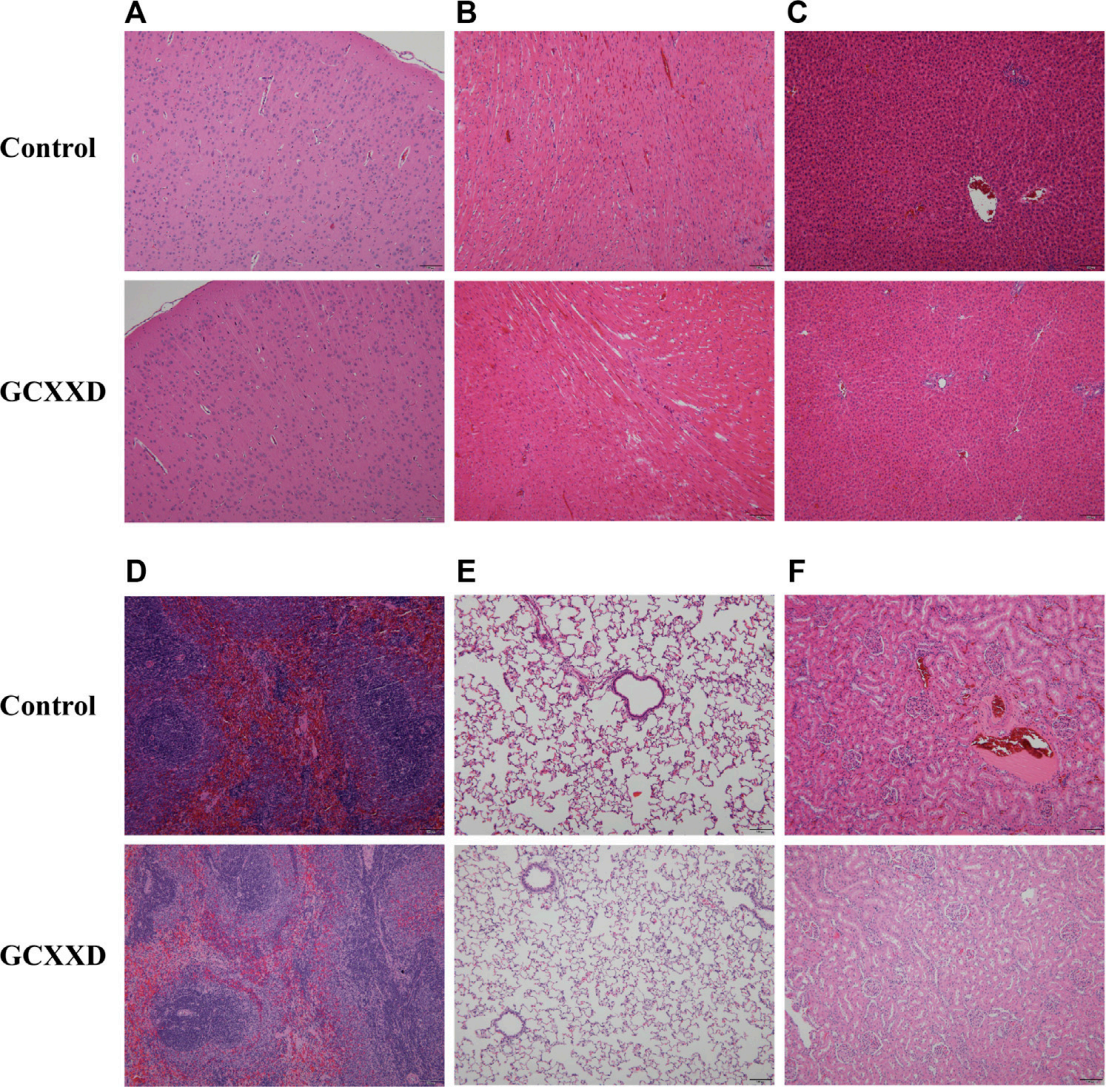
Representative histopathological microphotographs of the brain **(A)**, heart **(B)**, liver **(C)**, spleen **(D)**, lung **(E)**, and kidney **(F)** of rats in the control and GCXXD high-dose groups at the end of administration. Scale bar: 100 μm.

The histopathological findings of rats in the control and high-dose groups after 28 days of administration are summarized in Table 5, and the representative pathological images of major organs (brain, heart, liver, spleen, lungs, and kidneys) are displayed in Figure 3. Various lesions, including myocardial focal inflammatory cell infiltration in heart, hepatocyte steatosis and small granuloma in liver, interstitial pneumonia in lung, interstitial inflammatory cell infiltration in kidney, and cortical cell vacuolization in adrenal gland, were observed in rats of control and high-dose groups at the end of administration, which were considered spontaneous pathological changes. Thus, no pathological changes in the organs or tissues could be attributed to administration with GCXXD.

**TABLE 5:** Histopathological findings of rats after 28 days of administration with GCXXD (*n* = 10).

Organ lesion	Control			High-dose		
	+	++	+++	+	++	+++
Myocardial focal inflammatory cell infiltration	1	/	/	2	/	/
Hepatocyte steatosis	3	/	/	3	/	/
Liver small granuloma	1	/	/	2	/	/
Renal interstitial inflammatory cell infiltration	2	/	/	1	/	/
Interstitial pneumonia	1	/	/	1	/	/
Adrenal cortical cell vacuolization	4	/	/	3	/	/

+, mild; ++, moderate; +++, severe; /, no finding.

## 4 Discussion

So far, there is no toxicological study report on GCXXD, and only one article was found on the acute and subacute toxicity of Banxia Xiexin decoction (BXXXD), which is similar to the composition of GCXXD (Sui, 1996). BXXXD in that article was composed of seven herbal materials, including *Pinellia ternata* (Thunb.) Breit., *Scutellaria baicalensis* Georgi, *Glycyrrhiza uralensis* Fisch., *Panaxginseng* C. A. Mey., *Zingiber officinale* Rosc., *Ziziphus jujuba* Mill., and *Coptis chinensis* Franch. (ratio 5: 2.5: 2.5: 2.5: 2.5: 2.5: 1.0). The toxicity results showed that a single oral administration of BXXXD at dose of 2 or 8 g/kg did not cause death and toxic reactions in Sprague-Dawley rats, and it also has no toxicity to rats after administration of BXXXD at doses of 0.125, 0.5, and 2 g/kg for 5 weeks. The current toxicity results of GCXXD were similar to those of BXXXD. In the present study, GCXXD was administered to rats by gavage three times within 24 h at a dose of 10 g/kg, i.e. the total dose was 30 g/kg, which did not affect mortality, clinical signs, body weight, or gross observation. Thus, no acute toxicity was found in rats treated with GCXXD up to 30 g/kg/day, and the median lethal dose (LD_50_) of acute oral administration of GCXXD in rats could be assumed to be higher than 30 g/kg/day. After 28-day repeated doses of GCXXD to rats, only the high dose (10 g/kg) may cause hematologic toxicity (mild anemia). There were no treatment-related findings in clinical observations, body weight, food consumption, hematology, serum biochemistry, organ index, and histopathology of rats at medium and low doses (3.83 and 1.47 g/kg). Based on these results, the no-observed-adverse-effect level (NOAEL) and lowest-observed-adverse-effect level (LOAEL) of GCXXD for 28 days in rats are considered to be 3.83 and 10 g/kg/day in rats, respectively.

The hematological system is sensitive to toxic substances and has a higher consistency of pharmaceutical toxicity to humans and animals (Olson et al., 2000). In the current subacute toxicity study, mild decreases in RBC, HGB, and HCT were observed in female rats treated with high dose of GCXXD. Furthermore, these alterations exceed our internal historical value range of normal rats. Given that the relevant MCV, MCH, MCHC, Ret, TBIL, and other indicators were within the normal range, as well as no abnormality was found in the bone marrow smear examination, the anemia of rats caused by high dose of GCXXD was simple and mild, which returned to normal after 28 days of drug withdrawal.

Gender difference is one of the important issues in drug research and development. Studies have shown that the reason for the gender difference in drugs is variations between men and women in body weight, plasma volume, gastric emptying time, plasma protein levels, cytochrome P450 activity, drug transporter function, excretion activity, and other factors (Gandhi et al., 2004; Franconi, et al., 2012). The interaction between estrogen and various enzymes or receptors may also weaken or enhance the effect of compatible drugs, even toxic side effects (Parkinson et al., 2004). Researchers have found gender differences in the toxic reactions of some Chinese herbal medicines or Chinese herbal formulas. For example, the Zhixue capsule, a commercially available Chinese patent medicine, has been reported to show more obvious hepatotoxicity in female rats, and the proportion of liver injury in women was more than that in men (Fan et al., 2018). Of note, the changes in hematological parameters occurred only in female rats in the present subacute toxicity study. Whether it is related to the short duration of treatment, relatively small number of animals, physiological differences between genders, gender differences in drug metabolism, etc., these conjectures are questionable and could be further investigated in future studies.

Chinese medicinal materials are unprocessed plants, animals and minerals from nature, which cannot be directly used in prescriptions. They must undergo physical and/or chemical pretreatment processes (Paozhi in Chinese) after harvest to be prepared into decoction pieces for the purpose of preservation, enhancing efficacy, or detoxification. These processes include sun drying, stir frying, roasting, honey frying, wine frying, soil frying, vinegar frying, steaming, fumigation, calcination, etc (Liu et al., 2015). Some traditional Chinese materials have good efficacy, but have certain toxicity, and must be processed to reduce their toxicity, so as to ensure safety in clinical use, such as vinegar baked *Corydalis yanhusuo* W. T. Wang (Chinese name: Yanhusuo) and rice fried *Mylabris phalerata* Pallas (Chinese name: Banmao). The Chinese Pharmacopoeia (version 2015) includes 10 highly toxic Chinese medicines, 42 toxic Chinese medicines, and 30 minor toxic Chinese medicines (Wu, 2022). *Pinellia ternata* (Thunb.) Breit. (Chinese name: Banxia) contained in GCXXD is one of the toxic Chinese herbal medicine. From ancient times to the present, it has been recorded in many of the classic Materia Medica books that *Pinellia ternata* (Thunb.) Breit. possesses some toxic effects, such as tingling in the throat and mouth and inducing abortion and vomiting. Recent studies suggest that the toxic ingredients of *Pinellia ternata* (Thunb.) Breit. are mainly the needle crystal and lectin protein, which cause hepatotoxicity, gastrointestinal toxicity, cardiac toxicity, etc (Huang et al., 2020). The method of detoxification of *Pinellia ternata* (Thunb.) Breit. is generally processed with ginger juice, alum, licorice, and lime (Peng et al., 2022). The processed product of *Pinellia ternata* (Thunb.) Breit. (Fa Banxia in Chinese) was used in this study. The toxicity of raw Pinellia ternata was greatly reduced after being processed with alum, licorice, and lime blocks in turn. Therefore, no apparent toxicity was observed in rats in the acute and subacute toxicity studies of GCXXD.

## 5 Conclusion

In summary, the acute toxicity study showed that GCXXD is safe in rats without any obvious toxicity *via* an oral dose of 30 g/kg/day (3 × 10 g/kg). In the subacute toxicity study, the NOAEL and LOAEL of oral administration of GCXXD for 28 days in rats are considered to be 3.83 g/kg and 10 g/kg, respectively, which are approximately equal to 21-fold and 55-fold of its proposed dosage in humans. Slightly decreased RBC, HGB, and HCT were observed in female rats at 10 g/kg, suggesting that repeated oral administration of high-dose GCXXD may cause mild anemia in female rats. Chronic toxicity studies in rodents should be carried out to further support the safe use of GCXXD.

## Data Availability

The original contributions presented in the study are included in the article/Supplementary Material, further inquiries can be directed to the corresponding author.

## References

[B1] Cai X. Gan L. (2022). Analysis on clinical application of Gancao Xiexin decoction. Jiangxi J. Traditional Chin. Med. 53, 32–34. (in Chinese).

[B2] Calapai G. (2012). Pharmacovigilance and herbal medicines. Eur. J. Integr. Med. 96, 96. 10.1016/j.eujim.2012.07.696

[B3] Efferth T. Kaina B. (2011). Toxicities by herbal medicines with emphasis to traditional Chinese medicine. Curr. Drug Metab. 12, 989–996. 10.2174/138920011798062328 21892916

[B4] Ekor M. (2014). The growing use of herbal medicines: Issues relating to adverse reactions and challenges in monitoring safety. Front. Pharmacol. 4, 177. 10.3389/fphar.2013.00177 24454289 PMC3887317

[B5] Fan Q. Zhao B. Zhang J. Li P. Jia X. Wang T. (2018). Analysis on drug factors for hepatotoxicity of Zhixue Capsule. Chin. J. Exp. Traditional Med. Formulae 24, 150–157. (in Chinese).

[B6] Farzaei M. H. Bayrami Z. Farzaei F. Aneva I. Das S. K. Patra J. K. (2020). Poisoning by medical plants. Arch. Iran. Med. 23, 117–127. 32061075

[B7] Franconi F. Campesi I. Occhioni S. Antonini P. Murphy M. F. (2012). Sex and gender in adverse drug events, addiction, and placebo. Handb. Exp. Pharmacol. 2012, 107–126. 10.1007/978-3-642-30726-3_6 23027448

[B8] Gandhi M. Aweeka F. Greenblatt R. M. Blaschke T. F. (2004). Sex differences in pharmacokinetics and pharmacodynamics. Annu. Rev. Pharmacol. Toxicol. 44, 499–523. 10.1146/annurev.pharmtox.44.101802.121453 14744256

[B9] He Y. Zou A. Tu Z. Liu H. (2022). Study on the consistency of HPLC fingerprint of formulated granules of Gancao Xiexin decoction and traditional decoction. Tianjin Pharm. 34, 14–21. (in Chinese).

[B10] Hu Y. Li J. Jiang M. Min Z. (2022). Progress in pharmacological research and clinical application of Gancao Xiexin Decoction. Pharm. Clin. Chin. Materia Medica 13, 80–84. (in Chinese).

[B11] Huang F. Gao J. Gong Q. (2020). Research progress on pharmacological effects and toxicity of Pinellia ternata. Nat. Prod. Res. Dev. 32, 1773–1781. (in Chinese).

[B12] Hudson A. Lopez E. Almalki A. J. Roe A. L. Calderon A. I. (2018). A review of the toxicity of compounds found in herbal dietary supplements. Planta Med. 84, 613–626. 10.1055/a-0605-3786 29672820

[B13] Li M. Liu X. Xu X. Li T. (2022). Anti inflammatory effect of Gancao Xiexin Decoction combined with Dragon's blood on DSS induced UC model rats. J. Shaanxi Univ. Chin. Med. 45, 77–83. (in Chinese).

[B14] Liu S. H. Chuang W. C. Lam W. Jiang Z. Cheng Y. C. (2015). Safety surveillance of traditional Chinese medicine: Current and future. Drug Saf. 38, 117–128. 10.1007/s40264-014-0250-z 25647717 PMC4348117

[B15] Luo Y. T. Wu J. Zhu F. Y. Wu J. Q. Wu P. Liu Y. C. (2022). Gancao Xiexin decoction ameliorates ulcerative colitis in mice via modulating gut microbiota and metabolites. Drug Des. Devel Ther. 16, 1383–1405. 10.2147/DDDT.S352467 PMC9114650 35601674

[B16] Olson H. Betton G. Robinson D. Thomas K. Monro A. Kolaja G. (2000). Concordance of the toxicity of pharmaceuticals in humans and in animals. Regul. Toxicol. Pharmacol. 32 (1), 56–67. 10.1006/rtph.2000.1399 11029269

[B17] Parkinson A. Mudra D. R. Johnson C. Dwyer A. Carroll K. M. (2004). The effects of gender, age, ethnicity, and liver cirrhosis on cytochrome P450 enzyme activity in human liver microsomes and inducibility in cultured human hepatocytes. Toxicol. Appl. Pharmacol. 199, 193–209. 10.1016/j.taap.2004.01.010 15364537

[B18] Peng W. Li N. Jiang E. Zhang C. Huang Y. Tan L. (2022). A review of traditional and current processing methods used to decrease the toxicity of the rhizome of Pinellia ternata in traditional Chinese medicine. J. Ethnopharmacol. 299, 115696. 10.1016/j.jep.2022.115696 36087845

[B19] Shen L. Luo K. Wen X. Wang S. Fan X. (2021). Systematic chemical characterization of Xiexin decoctions using high performance liquid chromatography coupled with electrospray ionization mass spectrometry. Chin. J. Nat. Med. 19, 464–472. 10.1016/S1875-5364(21)60045-6 34092297

[B20] Shi K. Zhang Q. (2022). Prescription research and clinical application of Gancao Xiexin decoction. J. Liaoning Univ. TCM 24, 89–96. (in Chinese).

[B21] Sui F. (1996). Experimental study on oral toxicity of Banxia Xiexin decoction. Foreign Med. J. Traditional Chin. Med. 18, 46. (in Chinese).

[B22] Tang H. Zhai S. Chen C. Yan Z. Zhu M. (2022). Simultaneous determination of eight constituents in Gancao Xiexin decoction by QAMS. Chin. Tradit. Pat. Med. 44, 1410–1415. (in Chinese).

[B23] Wang J. Wong Y. K. Liao F. (2018). What has traditional Chinese medicine delivered for modern medicine? Expert Rev. Mol. Med. 20, e4. 10.1017/erm.2018.3 29747718

[B24] World Health Organization (Who) (2008). Traditional medicine. Fact SheetArchived Orig. 28.07.08 134, 2003–2005.

[B25] Wu T. Gao Y. Y. Su J. Tang X. N. Chen Q. Ma L. W. (2022). Three-dimensional bioprinting of artificial ovaries by an extrusion-based method using gelatin-methacryloyl bioink. Chin. Traditional Med. Sci. Technol. 29, 170–178. (in Chinese). 10.1080/13697137.2021.1921726 33993814

[B26] Wu W. Huang M. Xu H. (2021). Effect of Gancao Xiexin Decoction combined with Kangfuxin Liquid treatment on symptom scoreimmune function and adverse reactions for patients with ulcerative proctitis. J. Sichuan Traditional Chin. Med. 40, 107–110. (in Chinese).

[B27] Xu W. Zhu L. Zu L. Yan S. Zhang H. Dou D. (2020). Formulation and consideration of World Health Organization international classification of traditional medicine. J. Tradit. Chin. Med. 40, 157–161. 32227778

[B28] Yan J. Yan Y. Young A. Yan Z. Yan Z. (2021). Effectiveness and safety of Chinese medicine decoctions for behcet's disease: A systematic review and meta-analysis. Evid. Based Complement. Altern. Med. 2021, 8202512. 10.1155/2021/8202512 PMC8313333 34335839

[B29] Yuan H. Ma Q. Ye L. Piao G. (2016). The traditional medicine and modern medicine from natural products. Molecules 21, 559. 10.3390/molecules21050559 27136524 PMC6273146

[B30] Zeng L. H. Wu Z. F. Wang F. Wang X. C. Wang Y. Q. Yue P. F. (2017). Status, problems and warranty strategy of quality uniformity for traditional Chinese medicine preparations. Zhongguo Zhong Yao Za Zhi 42, 3826–3830. 10.19540/j.cnki.cjcmm.20170907.013 29235302

[B31] Zhang J. Chen Q. Chen Y. (2021). Study on the mechanism of Gancao Xiexin decoction in treating ulcerative colitis in rats by TLR4/NF-κB signal pathway. Fujian J. TCM 52, 22–24. (in Chinese).

[B32] Zhang J. Yu Y. Xu S. Li J. Yin S. Jian W. (2021). A meta analysis of modified licorice heart-draining decoction in the treatment of recurrent aphthous ulcer. Henan Tradit. Chin. Med. 41, 997–1002. (in Chinese).

